# Plasma lipids and glycaemic indices in Australians following plant-based diets versus a meat-eating diet

**DOI:** 10.1186/s12944-024-02340-5

**Published:** 2024-10-26

**Authors:** Grace Austin, Jessica J. A. Ferguson, Shaun Eslick, Christopher Oldmeadow, Lisa G. Wood, Manohar L. Garg

**Affiliations:** 1 School of Biomedical Sciences & Pharmacy, University of Newcastle, Callaghan, NSW Australia; 2 Food and Nutrition Research Program, Hunter Medical Research Institute, New Lambton Heights, NSW Australia; 3 School of Health Sciences, University of Newcastle, Callaghan, NSW Australia; 4 Macquarie Medical School, Macquarie University, Level 1, 75 Talavera Road, Macquarie Park, NSW 2109 Australia; 5 Clinical Research Design, Information Technology and Statistical Support Unit, Hunter Medical Research Institute, New Lambton, NSW Australia

**Keywords:** Lipids, Insulin, Glucose, Cardio-vascular disease, Plant-based, Vegan, Vegetarian, T2D; dyslipidaemia; pesco-vegetarian; semi-vegetarian

## Abstract

**Background:**

Vegan and vegetarian dietary patterns are known to beneficially modulate risk factors for cardiovascular disease; however, the current literature does not differentiate between various plant-based diets. This study aimed to examine the association between various plant-based diets and plasma lipids and glycaemic indices compared to a regular meat-eating diet.

**Methods:**

A cross-sectional study of Australian adults (*n* = 230) aged 30-75yrs habitually consuming the following were recruited: vegan, lacto-vegetarian, pesco-vegetarian, semi-vegetarian, or regular meat-eater. Multivariable regression analysis was used to adjust for covariates.

**Results:**

Compared to regular meat-eaters, vegans had significantly lower total cholesterol (-0.77mmol/L,95% CI -1.15, -0.39, *P* < 0.001), low-density lipoprotein cholesterol (LDL-C, -0.71mmol/L, 95% CI -1.05, -0.38, *P* < 0.001), non-high-density lipoprotein cholesterol (non-HDL-C, -0.75mmol/L, 95% CI -1.11, -0.39, *P* < 0.001), total cholesterol/HDL-C-ratio (-0.49mmol/L, 95% CI -0.87, -0.11, *P* = 0.012), fasting blood glucose (FBG, -0.29mmol/L, 95% CI -0.53, -0.06, *P* = 0.014), haemoglobin A1C (-1.85mmol/mol, 95% CI -3.00, -0.71, *P* = 0.002) and insulin (-1.76mU/L, 95% CI -3.26, -0.26, *P* = 0.021) concentrations. Semi-vegetarians had significantly lower LDL-C (-0.41mmol/L, 95% CI -0.74, -0.08, *P* = 0.041) and non-HDL-C (-0.40mmol/L, 95% CI -0.76, -0.05, *P* = 0.026) and lacto-ovo vegetarians had significantly lower FBG (-0.34mmol/L, 95% CI -0.56, -0.11, *P* = 0.003) compared to regular meat-eaters. There were no differences in HDL-C and triglycerides between plant-based and regular-meat diets.

**Conclusions:**

Plasma lipaemic and glycaemic measures as a collective were more favourable among vegans, whereas among lacto-ovo vegetarians and semi-vegetarians, only some measures were favourable.

**Trial registration:**

ACTRN12621000743864. Date 6/11/2021.

**Supplementary Information:**

The online version contains supplementary material available at 10.1186/s12944-024-02340-5.

## Background

In 2022, it was estimated that over 1.3 million Australians were diagnosed with type 2 diabetes mellitus (T2D) [1] and over 2.1 million reported hyperlipidaemia [2]. These chronic, non-communicable, progressive conditions are independent contributors to the onset of cardiovascular disease (CVD), which represented close to a third of all deaths globally in 2021 [3]. Lipids play a vital role in the development of atherosclerosis, a leading cause of CVD [4]. A lipid profile characterised by elevated total cholesterol (TC), triglycerides (TG), low-density lipoprotein (LDL-C) and reduced high-density lipoprotein (HDL-C), is widely recognised in the assessment of atherosclerosis and management of CVD [4]. Sustained dyslipidaemia leads to lipids and fibrous elements to build up in arteries resulting in the formation of atheroma plaques which causes narrowing of the arteries thus increasing risk of various CVDs and cardio-metabolic related conditions [5]. Individuals with T2D also have considerably higher risk of CVD morbidity and mortality [6]. Haemoglobin A1C (HbA1c) is the most used biomarker for diagnosis of T2D and pre-diabetes, although fasting blood glucose (FBG) and insulin also play an important role in marking disease progression and monitoring disease management. T2D and elevated glycaemic indices predisposes individuals to atherosclerotic CVD through pathogenic mechanisms mainly linked to hyperglycaemic and sustained chronic hyperinsulinemia which disrupts metabolic profiles via intracellular signalling pathways [7].

Elevated plasma lipids and glycaemic indices are caused by a complex interaction of environmental, genetic and lifestyle factors [8]. Key management strategies include modifications to lifestyle factors such as dietary pattern and physical activity as they influence numerous metabolic pathways involved in disease prognosis and progression including lipid metabolism, insulin utilisation, blood pressure and body composition [8]. Glucose and lipid metabolism are closely linked to each other given their important roles in energy metabolism. Studies have shown hypertriglyceridemia and low concentrations of HDL-C may not only be the consequence but also the cause of disturbed glucose metabolism [9]. Therefore, it is essential to uncover dietary interventions that target both these metabolic pathways to reduce risk of CVD and other cardiometabolic related morbidities.

The adoption of plant-based diets (PBDs) has become a global movement with recent market research indicating 12% of the Australian and United Kingdom populations are following a vegetarian or minimal meat diet [10, 11]. PBDs may be linked to favourable nutrient compositions with lower reported dietary intakes of saturated fat and cholesterol and higher intakes of dietary fibre, unsaturated fats, and polyphenols [12, 13]. If planned carefully to provide adequate nutrition they have been shown to be superior in comparison to a regular meat-eating diet for various health outcomes such as reduced risk of CVD [14], T2D [15] weight loss [16, 17]. A broad definition of PBDs within the scientific community is a dietary pattern high in vegetables, fruit, whole grains, legumes, nuts and unsaturated oil, and nil or low intakes of meat, poultry and/or seafood [14]. In order to create standardised definitions of PBDs, this study will use definitions previously implemented in Australian cohorts [18–21] which was originally adapted from Mihrshahi et al. [22] and aligned with the World Health Organisation [23]. These definitions focus on PBDs that typically exclude meat, dairy, and seafood consumption to various degrees (vegan, lacto-ovo vegetarian, pesco-vegetarian and semi-vegetarian).

PBDs have been offered as a potential strategy for managing glycaemic control [24] and plasma lipids [25]. A systematic review of five randomised controlled trials (RCTs), reported a significant improvement in markers of glycaemic control from baseline to end-point after at least four weeks or more of PBD intervention. In this review, PBD interventions were not defined in detail and vegan and vegetarian dietary patterns were pooled together in the analyses [26]. Another systematic review of 71 studies which were mostly cross-sectional, found vegetarian dietary patterns (including vegans) were associated with lower FBG compared to a omnivorous diet [27]. Studies included in this review also vaguely defined vegan and vegetarian diets or included no definition, and all studies reported a high risk of bias, therefore, differences between variations of plant-based eating were not established. Very few studies have investigated the effects of PBDs, inclusive of pesco-vegetarian and semi-vegetarian on glycaemic indices and risk of T2D. The Adventist Health-2 study (AHS-2) found the vegans, lacto-ovo vegetarians, semi-vegetarians but not pesco-vegetarians were associated with a substantial and independent reduced risk of T2D after a two year follow up period [28]. There has only been one analysis on an Australian cohort that evaluated glycaemic indices and risk of T2D among individuals following various PBDs, which was a secondary analysis among older women [15]. Prevalence of impaired glucose tolerance was reported to be lower in vegans, vegetarians, semi-vegetarians, and pesco-vegetarians compared to regular meat-eaters [15].

The effect of PBDs has also proved to be advantageous in the control of plasma lipids. Multiple pooled meta-analyses of both RCTs and observational studies have reported that compared to RMEs, individuals following vegan and vegetarian diets demonstrate lower concentrations of TC, LDL-C and HDL-C [25, 29–31]. Despite lower concentrations of HDL-C reported in observational studies, vegetarian diets have not been associated with poor cardiovascular health [32] and genetic variants that raise HDL-C do not necessarily reduce risk of coronary heart disease [33]. The AHS-2 study included analyses of additional categories of plant-based eating and found the risk of elevated TC and LDL-C beyond the recommended ranges were lower in lacto-ovo, pesco-vegetarians and vegans, although semi-vegetarians were not examined in this sample [34]. There has not been a previous Australian-based study which had the primary aim to evaluate the association between plasma lipids and various types of PBDs. Given the growing popularity of PBDs, an Australian-based study specifically designed to investigate individuals habitually following PBDs is warranted to understand the potential nutritional implications of PBDs and their influence on important cardiometabolic parameters.

Vegetarian diets (including vegans) have been associated with lower glycaemic and plasma lipids; however, previous literature does not explore various types of commonly adhered to PBDs in society and lacks cohesive categorisation. Therefore, the aim of this cross-sectional study is to examine the association between individuals following various PBDs and plasma lipids and glycaemic indices compared to a regular meat-eating diet.

## Materials and methods

### Study design and participants

The research protocol detailing the study design has been published elsewhere [20]. This cross-sectional study of 230 aged 30–75 years, involved data collection at one time point and was conducted at the Nutraceuticals Research Program, School of Biomedical Sciences & Pharmacy, University of Newcastle, Callaghan NSW, Australia. Those aged 30–75 following a dietary pattern for ≥ 6 months were eligible to participate. Participants that were ineligible were pregnant or breast feeding, had current or previous diagnosis of CVD, and/or significantly changed their usual physical activity levels or dietary pattern within the last 6 months. Additional exclusions applied to those taking lipid medications and/or glycaemic agents. The assessment of habitual weekly intake of meat, seafood, eggs, and dairy was used as an screening criteria to categorise participants into dietary patterns and has been published elsewhere [20]. The dietary patterns in which participants were recruited into include, vegan (nil animal-based foods), lacto-ovo vegetarian (LOV, nil meat, ± eggs, ± dairy), pesco-vegetarian (PV, nil meat, seafood consumption ≥ 1 per week, ± dairy, ± eggs), semi-vegetarian (SV, meat consumption ≤ 2 per week) or regular meat-eaters (RMEs) (meat consumption ≥ 7 per week). Lead investigator collected two-day diet histories and fasted blood samples via venepuncture after an overnight fast (~ 10–12 h) from enrolled participants who provided written informed consent. A self-reported questionnaire was completed by participants to collect information regarding demographic and medical history, prescribed or over-the-counter medication(s), habitual supplement use, smoking status, duration of following dietary pattern, level of education, age, and sex. Definitions for overweight and obese were; BMI ≥ 25 kg/m^2^ and ≥ 30 kg/m^2^, respectively [35].

### Assessment of dietary patterns

The Food Frequency Questionnaire (FFQ) employed to evaluate food and nutrient intake over the previous 3–6 months was the online self-administered 120-question Australian Eating Food Survey (AES^®^) FFQ which has been validated in the Australian population [36, 37]. Qualitative intake of food groups and food categories was derived from the total sum of all food frequency questions relating to the food group. An Accredited Practicing Dietitian conducted a comprehensive in-person interview to collect diet history across two habitual days and quantitatively evaluate the usual eating patterns of participants. Data was presented as average means consumed (mg/g) per day from micronutrients and macronutrients. Intake of plant-based dairy and meat alternatives and food fortification were included in the data collection. Assessment of micronutrient and macronutrients dietary intake is more accurately obtained through dietitian administered diet histories than FFQs [38]. FoodWorks (version 10, Xyris^®^, Brisbane, Australia, sourced online) was used to assess data.

### Assessment of plasma lipids and glycaemic indices

Blood lipids collected included TC, TG, LDL-C, HDL-C, non-HDL-C and TC/HDL-C ratio. Glycaemic indices collected included FBG, HbA1c and insulin. All samples were collected by a trained phlebotomist via venepuncture after an overnight fast (~ 10–12 h) on the same day as the study appointment which were measured using an auto-analyser by the commercial pathology service provider, NSW Health Pathology. Additional analyses were also conducted to evaluate prevalence of metabolic syndrome (MetS), metabolic components and Homeostatic Model Assessment for Insulin Resistance (HOMA-IR). The definition of MetS used was devised by the National Cholesterol Education Program Adult Treatment Panel III in 2001 and updated by the American Heart Association and the National Heart Lung and Blood Institute in 2005 [39]. This criteria of MetS includes having at least three of the following: abdominal obesity (> 102 cm for men and > 88 cm for women), hypertriglyceridemia (≥ 1.69 mmol/L), low HDL-C (< 1.03 mmol/L for men and < 1.29 mmol/L for women), hypertension (≥ 130/≥85mmHg), and elevated FBG (≥ 5.6 mmol/L). Participants taking antihypertensive, oral blood glucose and/or lipid lowering medications were considered to indicate the presence of the respective risk factor. HOMA-IR was used to quantify insulin resistance and beta-cell function. It was calculated by multiplying insulin FBG, then dividing by the constant 22.5, i.e. HOMA-IR = (insulin×FBG)/22.5 [40]. A HOMA-IR of ≥ 1.7 was used as the cut off for insulin resistance [41–43].

### Covariate analyses

Self-reported questionnaires were used to collect demographic characteristics (age, sex, education, duration of dietary pattern) and lifestyle factors (smoking status, physical activity, supplement, and medication use). The remaining variables were collected from a two-day dietitian administered diet history (total energy intake and alcohol consumption). The validated International Physical Activity Questionnaire (IPAQ, Long Version October 2002) was used to evaluate daily physical activity levels which was expressed as metabolic equivalent of task minutes per week (METs/week) [44]. Smoking status was categorised as ‘non-smoker’ or ‘current smoker’. Height was collected to the nearest 0.5 cm and weight to the nearest 0.1 kg by a qualified and certified clinician which was used to calculate BMI (kg/m^2^). Models were adjusted for age, sex, physical activity, energy intake, duration of dietary pattern, alcohol intake, smoking status, level of education, BMI as a mediator and the addition of eicosapentaenoic acid (EPA)/docosahexaenoic acid (DHA) supplement use in the analyses of plasma lipids.

### Statistical analyses

Normality of the participant characteristics data was assessed for normal distribution via inspection of histograms and quantile plot and reported as mean ± SD and categorical data as counts (n) and frequencies (%). Participant characteristics and nutrient intake levels were compared across dietary pattern groups via one-way ANOVA and Tukey post-hoc test or Fisher’s Exact tests. Under the assumption that a mean difference in LDL/HDL ratio between RMEs and vegans is 0.6mmol/L [45] (a significant and clinically relevant difference) and between subject standard deviation is 0.9mmol/L, it was estimated that a sample size of 36 per group was required to elicit a power of 80% to detect a difference (Cohens D 0.6 SD) at the confidence interval of 95%. A total of 44–48 participants were recruited into each dietary group. To identify the association between various PBDs and lipaemic and glycaemic biomarkers compared to a regular meat-eating diet, a Seemingly unrelated regression model was employed on each outcome to appropriately adjust for the mediating effect of BMI as well as other important confounders. Variables included in the adjusted models included age, sex, physical activity, energy intake, duration of dietary pattern, alcohol intake, smoking status, level of education, BMI as a mediator and the addition of EPA/DHA supplement use in the analyses of plasma lipids [46]. The regular-meat diet was considered as the reference group and PBDs as the exposure variable. Additional diagnostic procedures for identification of potential confounders involved the inspection of residual plots for normality and homogeneity. A sensitivity analysis was performed to assess how robust the results were to the previously mentioned possible confounders by comparing E-values to beta-coefficients. Significance level was calculated via Bonferroni corrected alpha *P* < 0.001 (*n* = 10 tests) and *P* < 0.05 were also reported. For appropriate graphical comparison between PBDs and a regular meat-eating diet, adjusted *β* coefficients ± SD of the significant variables are presented as figures. Percentage difference was calculated by using the adjusted mean difference between the exposure and reference group, divided by the reference group, multiplied by 100. Statistical analyses was conducted using StataCorp, 2016 (Stata Statistical Software: Release 17, College Station, TX, USA: StataCorp LP).

## Results

### Participant characteristics

In total 230 participants were included in this cross-sectional study. The mean age of participants was 54 ± 10 years, two thirds were female, just under half (40%) were overweight or obese, 6% were smokers, 80% had a higher education, and physical activity levels were comparable across groups (Table 1). Vegans were significantly younger and had a shorter duration of following dietary patterns in comparison to RMEs. 11% of the sample supplemented with omega-3 poly-unsaturated fatty acids (n-3 PUFAs), majority were EPA/DHA based, and reported use was higher among RMEs compared to those adhering to a PBD. Prevalence of MetS, individual metabolic components and HOMAR-IR were not significantly different between plant-based and regular-meat diets, except for elevated FBG which was higher among regular-eating diets compared to PBDs.

**Table 1:** Characteristics of participants across different dietary pattern groups

	Total sample (*n* = 230)	Vegan (*n* = 48)	Lacto-ovo vegetarian (*n* = 47)	Pesco-vegetarian (*n* = 46)	Semi-vegetarian (*n* = 44)	Regular meat eater (*n* = 45)	*P*
Women	177 (78.0%)	34 (77.0%)	36 (%76.6)	37 (80.4%)	36 (81.8%)	34 (75.6%)	0.754
Age (years)	53.5 ± 10.3	47.8 ± 10.0^a^	53.4 ± 9.9^b^	55.9 ± 11.2^b^	54.8 ± 8.8^b^	55.9 ± 9.7^b^	< 0.001
BMI (kg/m^2^)	24.3 ± 4.1	24.5 ± 4.1	24.4 ± 3.3	25.2 ± 4.6	23.9 ± 4.8	23.9 ± 3.5	0.559
Overweight or obese	93 (40.4%)	17 (35.4%)	23 (48.0%)	18 (39.1%)	13 (29.5%)	22 (48.9%)	0.248
Higher education	203 (88.3%)	42 (87.5%)	40 (85.1%)	42 (91.3%)	37 (84.1%)	42 (99.3%)	0.599
Physical Activity (METs)	5326 ± 5326	5775 ± 4036	6972 ± 8888	4945 ± 4119	4395 ± 3619	5163 ± 3671	0.179
Current Smoker	13 (5.7%)	5 (10.4%)	3 (6.4%)	1 (2.2%)	2 (4.6%)	2 (4.4%)	0.557
Dietary pattern duration (yrs)	16.6 + 17.9	6.8 ± 7.7^a^	16.3 ± 13.8^b^	16.2 ± 14.8^b^	11.9 ± 14.1^b^	32.4 ± 24.4^c^	< 0.001
**Supplement use (count (%))** ^**d**^							
N-3 PUFAs	25 (10.9)	3 (6.3)	7 (14.9)	4 (8.7)	2 (4.6)	9 (20.0)	0.117
EPA/DHA	22 (9.6%)	2 (4.2)	5 (10.6)	4 (8.7)	2 (4.6)	9 (20.0)	0.099
ALA	3 (1.3)	1 (2.1)	2 (4.3)	0	0	0	0.488
**Metabolic syndrome and components (count (%))** ^**e**^							
Metabolic syndrome	14 (6.1)	2 (4.2)	2 (4.2)	3 (6.5)	4 (9.1)	2 (4.4)	0.855
Abdominal obesity	74 (32.2)	12 (25.0)	19 (40.4)	15 (32.6)	9 (20.5)	19 (42.2)	0.113
Hypertriglyceridemia	31 (13.5)	6 (12.5)	9 (19.2)	5 (10.9)	7 (15.9)	4 (8.9)	0.638
Low HDL-C	29 (12.6)	11 (22.9)	5 (10.6)	6 (13.0)	5 (11.4)	2 (4.4)	0.122
Hypertension	22 (9.6)	2 (4.2)	4 (8.5)	5 (10.9)	3 (6.8)	8 (17.8)	0.266
Elevated FBG	13 (6.7)	1 (2.1)	0	3 (6.5)	3 (6.8)	6 (13.3)	0.042
HOMAR-IR	1.4 ± 1.4	1.2 ± 1.0	1.4 ± 1.0	1.3 ± 1.0	1.4 ± 1.0	1.5 ± 0.7	0.605
HOMAR-IR (cut off 1.7)	56 (24.4)	10 (20.8)	12 (25.5)	10 (21.7)	11 (25.0)	13 (28.9)	0.906

Data reported as means ± SD and for continuous variables and counts and (percentages) for categorical variables. Continuous data was compared using ANOVA and Tukey post-hoc test. Categorical data was compared using Fisher’s Exact

^a, b,c^Values within the same row without a common superscript letters are significantly different (*P* < 0.05)

^d^Participant is currently taking n-3 PUFA supplement as per medical history and were defined as; EPA/DHA (fish and krill based), ALA (algae and flaxseed based)

^e^The definition of metabolic syndrome and metabolic components are as per the National Cholesterol Education Program Adult Treatment Panel III further detailed in the methods section

ALA, Alpha-linolenic acid; BMI, body-mass index; EPA, eicosapentaenoic acid; FBG, fasting blood glucose; HbA1c, haemoglobin A1C; HOMA-IR, Homeostasis model assessment-estimated insulin resistance; DHA, docosahexaenoic acid; HDL-C, High-density lipoprotein cholesterol; IFFC, International Federation of Clinical Chemistry; MET, Metabolic equivalent of task minutes; NGSP, National Glycohemoglobin

### Nutrient analyses

Overall individuals adhering to a PBD, specifically vegans, had a more favourable nutrient composition compared to RMEs, characterised by significantly lower intake of saturated fats, trans fats, cholesterol, discretionary choices (including sugar sweetened beverages) and higher intake of PUFAs, dietary fibre, fruit and legumes/nuts compare to RMEs (Table 2). Individuals adhering to a PV dietary pattern had significantly higher seafood intake compared to all other groups and long chain n-3 PUFAs (LC n-3 PUFAs) compared to SVs, LOVs and vegans. Total energy intake and fat as a percentage of total energy intake (EN%) was comparable across dietary patterns and RMEs had a higher protein intake (EN%) compared to those following a PBD. In comparison to RMEs, carbohydrate (EN%) and dietary fibre intake were significantly higher in those following a vegan, LOV, and SV dietary pattern, additionally, dietary fibre intake of vegans was almost two times that of RMEs. Sugar intake was comparable, although intake of starch was significantly higher in vegans compared to RMEs.

**Table 2:** Dietary intake across dietary pattern groups derived from an average of two dietitian-administered diet histories (quantitative data) and the AES^®^ FFQ (qualitative data)

Nutrient/food group serves (per/day)	Total sample (*n* = 230)	Vegan (*n* = 48)	Lacto-ovo vegetarian (*n* = 47)	Pesco-vegetarian (*n* = 46)	Semi-vegetarian (*n* = 44)	Regular meat-eater (*n* = 45)	*P*
**Quantitative data**							
Energy (kJ)	9584 ± 2691	9957 ± 2712	9767 ± 3337	9041 ± 2325	9651 ± 2598	9489 ± 2361	0.545
Protein (%)^e^	16.5% ± 4.1	15.4% ± 3.6^a, b^	14.5% ± 4.0^a^	17.0% ± 3.1^b^	15.4% ± 2.9^a, b^	20.4% ± 4.3^c^	< 0.001
Carbohydrate (%)^e^	40.1% ± 9.3	43.9% ± 8.4^a^	41.7% ± 8.6^a, b^	37.8% ± 9.1^b, c^	41.9% ± 8.3^a, b^	35.0% ± 9.8^c^	< 0.001
Sugar (g)	86.0 ± 38.2	77.3 ± 26.4	92.4 ± 46.7	82.2 ± 32.4	92.0 ± 29.6	88.3 ± 49.8	0.241
Starch (g)	147.5 ± 76.9	184.7 ± 86.9^a^	153.2 ± 79.1^a, b^	125.6 ± 55.8^b^	155.4 ± 76.9^a, b^	116.5 ± 64.0^b^	< 0.001
Total fat (%)^e^	37.4% ± 8.2	35.2% ± 8.4	38.1% ± 8.4	38.7% ± 7.8	36.9% ± 7.0	38.1% ± 8.9	0.236
Saturated fat (g)	28.5 ± 13.3	23.3 ± 11.2^a^	28.0 ± 14.3^a, b^	27.8 ± 13.2^a, b^	30.7 ± 11.5^a, b^	33.1 ± 14.6^b^	0.006
Saturated fat (%)^e^	11.0% ± 4.1	8.7% ± 4.1^a^	10.6% ± 4.1^a^	11.4% ± 4.1^b^	11.8% ± 3.2^b^	12.8% ± 3.6^b^	< 0.001
Trans fats (g)	0.9 ± 0.6	0.5 ± 0.7^a^	0.8 ± 0.5^a^	1.0 ± 0.6^b^	1.0 ± 0.6^b^	1.3 ± 0.6^b^	< 0.001
MUFAs (g)	39.0 ± 15.3	37.0 ± 15.6	40.0 ± 15.9	40.3 ± 16.3	38.1 ± 14.8	39.8 ± 14.2	0.806
PUFAs (g)	20.3 ± 10.4	25.8 ± 13.6^a^	21.3 ± 10.1^a, b^	18.5 ± 7.5^b^	19.2 ± 10.0^b^	16.2 ± 6.9^b^	< 0.001
LCn-3 PUFAs (g)	0.5 ± 0.7	0.0 ± 0.0^a^	0.0 ± 0.0^a, b^	1.0 ± 0.8^c^	0.4 ± 0.7^d^	0.8 ± 0.8^c^	< 0.001
ALA (g)	2.6 ± 2.4	3.7 ± 3.4^a^	2.8 ± 2.1^a, b^	2.1 ± 1.3^b^	2.6 ± 3.0^a, b^	1.6 ± 0.9^b^	< 0.001
n-6 PUFAs (g)	17.5 ± 8.8	22.3 ± 10.7^a^	19.1 ± 8.8^a^	15.5 ± 6.9^b^	16.8 ± 8.2^b^	13.6 ± 6.1^b^	< 0.001
Cholesterol (mg)	142.3 ± 138.4	33.6 ± 92.1^a^	94.8 ± 122.5^a, c^	160.7 ± 108.7^b^	152.4 ± 112.6^b, c^	279.1 ± 124.8^d^	< 0.001
Dietary fibre (g)	45.9 ± 20.9	58.0 ± 18.8^a^	51.6 ± 29.7^a^	41.5 ± 13.4^b, c^	45.6 ± 16.7^b^	31.8 ± 9.4^c^	< 0.001
Alcohol (g)	5.1 ± 10.0	1.8 ± 4.3^a^	3.5 ± 7.2^a^	6.7 ± 11.4^a, b^	4.0 ± 8.1^a, b^	9.4 ± 14.4^b^	0.002
**Qualitative data**							
Vegetables	5.9 ± 2.4	6.3 ± 2.5	6.2 ± 2.7	5.9 ± 2.1	6.0 ± 2.1	5.1 ± 2.7	0.126
Non-Starchy	4.9 ± 2.1	5.3 ± 2.0	5.0 ± 2.2	4.9 ± 1.9	5.0 ± 1.8	4.1 ± 2.5	0.103
Grains	2.7 ± 1.3	2.7 ± 1.1	2.6 ± 1.3	2.9 ± 1.6	2.7 ± 1.2	2.6 ± 1.5	0.911
Non-refined	2.1 ± 1.1	1.1 ± 1.0	2.0 ± 1.1	2.3 ± 1.5	1.9 ± 0.9	2.0 ± 1.2	0.599
Fruit	3.1 ± 1.7	3.8 ± 2.1^a^	3.0 ± 1.3^a.b^	3.0 ± 1.6^a, b^	3.2 ± 1.5^a, b^	2.7 ± 1.6^b^	0.023
Protein-rich foods^f^	1.9 ± 0.9	1.5 ± 0.7^a^	1.3 ± 0.5^a^	2.0 ± 0.6^b^	1.9 ± 1.0^a, b^	2.7 ± 0.7^c^	< 0.001
Legumes/nuts	1.1 ± 0.6	1.5 ± 0.7^a^	1.1 ± 0.5^b^	1.1 ± 0.5^b^	1.0 ± 0.5^b^	0.6 ± 0.5^c^	< 0.001
Seafood	0.2 ± 0.3	0.0 ± 0.0^a^	0.0 ± 0.0^a^	0.6 ± 0.4^b^	0.2 ± 0.4^c^	0.3 ± 0.2^c^	< 0.001
Red meat	0.2 ± 0.4	0.0 ± 0.0^a^	0.0 ± 0.0^a^	0.0 ± 0.0^a^	0.2 ± 0.2^b^	1.0 ± 0.5^c^	< 0.001
Dairy	1.4 ± 1.4	0.0 ± 0.0^a^	1.6 ± 1.5^b^	1.9 ± 1.0^b^	1.7 ± 1.2^b^	2.0 ± 1.4^b^	< 0.001
Discretionary choices	1.7 ± 1.2	1.3 ± 1.0^a^	1.6 ± 1.1^a^	1.5 ± 1.1^a^	1.8 ± 1.2^a, b^	2.2 ± 1.5^b^	0.008
Sugar sweetened beverages	0.4 ± 0.6	0.3 ± 0.4^a^	0.3 ± 0.4^a^	0.3 ± 0.4^a^	0.3 ± 0.7^a, b^	0.6 ± 0.8^b^	0.024

Data are reported as absolute means ± SD and *p*-values reported from ANOVA and Tukey post-hoc test for pairwise comparisons

^a, b,c, d^Values within a row without a common superscript letters are significantly different (*P* < 0.05)

^e^Presented as % contribution of total energy intake

^f^Protein-rich foods include meats, poultry, seafood, eggs, legumes, and nuts. Assessment of meat exclusion among vegans and LOVs and dairy exclusion among vegans were derived from diet histories

ALA, α-linolenic acid; eq, equivalent; MUFAs, monounsaturated fatty acids; PUFAs, polyunsaturated fatty acids; LC n-3 PUFAs, long chain omega-3 polyunsaturated fatty acids; n-6 PUFAs, omega-6 polyunsaturated fatty acids

### Plasma lipids

**Fig. 1:**
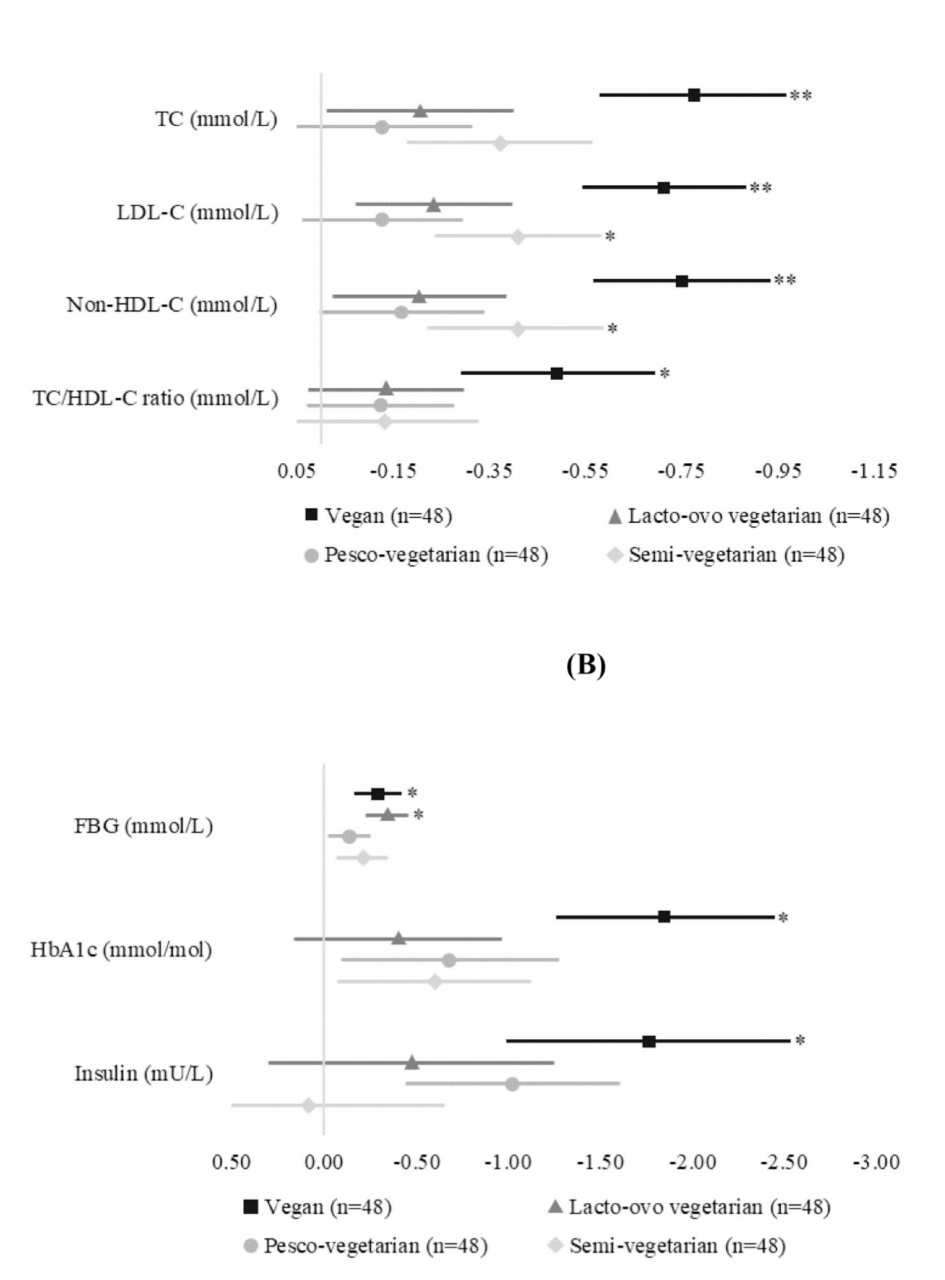
Adjusted mean differences ± SE in (a) lipid levels and (b) glycaemic indices of plant-based compared to regular meat-eating diets

FBG, fasting blood glucose; HbA1c, haemoglobin A1C; HDL-C, high-density lipoprotein cholesterol; LDL-C, low-density lipoprotein cholesterol; TC, total cholesterol; TG, triglycerides. *P < 0.05, **P = 0.001 (Bonferroni corrected alpha).

After adjustments, and compared to RMEs, the vegan dietary pattern was associated with significantly lower plasma lipids (Table 3; Fig. 1). Compared to RMEs, vegans had significantly lower TC by 14.9% (-0.77mmol/L,95% CI -1.15, -0.39, *P* < 0.001), LDL-C by 22.6% (-0.71mmol/L, 95% CI -1.05, -0.38, *P* < 0.001), non-HDL-C by 20.7% (-0.75mmol/L, 95% CI -1.11, -0.39, *P* < 0.001) and TC/HDL-ratio by 14.3% (-0.49mmol/L, 95% CI -0.87, -0.11, *P* = 0.012). In addition, SVs had significantly lower LDL-C by 12.4% (-0.41mmol/L, 95% CI -0.74, -0.08, *P* = 0.041) and non-HDL-C by 10.8% (-0.40mmol/L, 95% CI -0.76, -0.05, *P* = 0.026). Those following a LOV and PV dietary pattern did not have significantly different plasma lipids compared to RMEs. HDL-C and TG concentrations were comparable across plant-based and regular-meat dietary patterns. Supplementary Table 1 shows adjusted means, and 95% CIs used to calculate percentage differences in plasma lipids between PBDs and the regular meat-eating diet.

**Table 3:** Adjusted mean differences in lipid and glycaemic indices across plant-based diets compared to a regular meat-eating diet

Variable	Vegan (*n* = 48)		Lacto-ovo vegetarian (*n* = 47)		Pesco-vegetarian (*n* = 46)		Semi-vegetarian (*n* = 44)		
	β (95% CI)	*P*	β (95% CI)	*P*	β (95% CI)	*P*	β (95% CI)	*P*	
Lipid lipids									
TC (mmol/L)	-0.77 (-1.15, -0.39)	< 0.001*	-0.20 (-0.58, 0.17)	0.282	-0.12 (-0.50, 0.26)	0.526	-0.37 (-0.74, 0.00)		0.052
TG (mmol/L)	-0.08 (-0.31, 0.14)	0.462	0.07 (-0.12, 0.26)	0.487	-0.09 (-0.30, 0.13)	0.435	-0.01 (-0.20, 0.19)		0.948
LDL-C (mmol/L)	-0.71 (-1.05, -0.38)	< 0.001*	-0.23 (-0.56, 0.09)	0.158	-0.13 (-0.45, 0.19)	0.439	-0.41 (-0.74, -0.08)		**0.014**
HDL-C (mmol/L)	-0.03 (-0.18, 0.12)	0.693	0.02 (-0.12, 0.16)	0.806	0.05 (-0.10, 0.20)	0.517	0.04 (-0.10, 0.19)		0.578
Non-HDL-C (mmol/L)	-0.75 (-1.11, -0.39)	< 0.001*	-0.20 (-0.54, 0.13)	0.236	-0.17 (-0.52, 0.19)	0.356	-0.40 (-0.76, -0.05)		**0.026**
TC/HDL-C ratio (mmol/L)	-0.49 (-0.87, -0.11)	**0.012**	-0.13 (-0.43, 0.17)	0.381	-0.12 (-0.44, 0.19)	0.448	-0.13 (-0.52, 0.26)		0.510
**Glycaemic indices**									
FBG (mmol/L)	-0.29 (-0.53, -0.06)	**0.014**	-0.34 (-0.56, -0.11)	**0.003**	-0.14 (-0.36, 0.09)	0.232	-0.21 (-0.48, 0.06)		0.134
HbA1c (IFFC, mmol/mol)	-1.85 (-3.00, -0.71)	**0.002**	-0.40 (-1.51, 0.71)	0.479	-0.68 (-1.84, 0.48)	0.249	-0.60 (-1.63, 0.43)		0.254
Insulin (mU/L)	-1.76 (-3.26, -0.26)	**0.021**	-0.47 (-2.00, 1.05)	0.542	-1.03 (-2.17, 0.11)	0.078	0.09 (-1.36, 1.55)		0.902

Data is presented as β coefficients (95% CIs) and *p*-values. Multivariate regression analyses was used to adjust the model for age (years), sex (female, male), physical activity level (MET/week), total energy intake (kJ/day), duration of dietary pattern (years), alcohol intake (g), smoking status (yes, no), level of education (higher education yes, no), BMI (kg/m^2^) as a mediator and the addition of EPA/DHA supplement use (yes, no) for lipid lipids. Bold values indicate statistical significance (*P* < 0.05). *P-*values marked with an asterisk meet the Bonferroni corrected alpha (*P* = 0.001)

EPA, eicosapentaenoic acid; DHA, docosahexaenoic acid; FBG, fasting blood glucose; HbA1c, haemoglobin A1C; HDL-C, high-density lipoprotein cholesterol; LDL-C, low-density lipoprotein cholesterol; TC, total cholesterol; TG, triglycerides

### Glycaemic indices

After adjustments, and compared to RMEs, the vegan dietary pattern was associated with significantly lower glycaemic indices for all parameters (Table 3; Fig. 1). Compared to RMEs, vegans had significantly lower FBG by 6.2% (-0.29mmol/L, 95% CI -0.53, -0.06, *P* = 0.014), HbA1c by 5.8% (-1.85mmol/mol, 95% CI -3.00, -0.71, *P* = 0.002) and insulin by 28.7% (-1.76mU/L, 95% CI -3.26, -0.26, *P* = 0.021). In addition, LOVs had significantly lower FBG by 7.3% (-0.34mmol/L, 95% CI -0.56, -0.11, *P* = 0.003). Those following a PV and SV dietary pattern did not have significantly different glycaemic indices compared to RMEs. Supplementary Table 1 shows adjusted means, and 95% CIs used to calculate percentage differences in glycaemic indices between PBDs and the regular meat-eating diet.

### Sensitivity analyses and multiple comparisons

After completing a sensitivity analyses, E-values were small and significant outcomes remained unchanged, indicating it was unlikely there was a missing confounder with an observed association and exposure. In addition, outcomes among vegans for TC, LDL-C, non-HDL-C and HbA1c met the Bonferroni corrected alpha of *P* = 0.001. This provides further evidence that the vegan dietary pattern is robustly associated with favourable plasma lipids and glycaemic indices when compared to RMEs.

## Discussion

This cross-sectional study of adults found adherence to a vegan dietary pattern was associated with favourable plasma lipids and glycaemic indices when compared to a regular-meat dietary pattern characterised by lower concentrations of TC, LDL-C, non-HDL-C, TC/HDL ratio, FBG, HbA1c and insulin. Those following other PBDs demonstrated a less pronounced association, although SVs had significantly lower LDL-C and non-HDL-C and LOVs had significantly lower FBG when compared to RMEs. There were no differences observed between any plant-based and regular meat-eating dietary patterns on HDL-C and TG concentrations. These outcomes withstood adjustments for potential confounders, sensitivity analyses and were somewhat robust to adjustment for multiple testing.

This study indicates that vegans have a favourable lipid profile compared to RMEs, except there were no differences in TG and HDL-C. Our results align with multiple pooled meta-analyses of both RCTs and observational studies which reported that when compared RMEs, LOVs (including vegans) reduced or had lower concentrations of TC, LDL-C, non-HDL-C, although reported no difference in TG [25, 29–31]. These studies also reported lower HDL-C among vegetarians (including vegans), and one study found vegans to have higher TG, dissimilar to the current results. Crude analyses revealed a slightly lower HDL-C among vegans, the difference was not significant when compared to RMEs. Large overseas cross-sectional studies with similar methodologies found vegan and vegetarian dietary patterns to have significantly lower HDL-C, LDL-C, TC, TC/HDL-C ratio, and higher TG [47, 48]. Reasons why current HDL-C concentrations do not align with previous literature may be attributed to the ‘healthy user effect’ as participants in the current sample had comparable levels of MetS and metabolic components including obesity and hypertension, known to influence plasma lipids [49]. To the best of our knowledge, no other study has investigated plasma lipids across four categories of plant-based eating. The AHS-2 is the only other study to explore dietary patterns and health parameters among vegans, LOVs and PVs and reported that the prevalence of elevated TC and LDL-C were lower in all three groups compared to non-vegetarians [34]. Noteworthy, among this study, individuals were Seventh Day Adventists, whom are known to also practice other health-promoting behaviours such as abstaining from alcohol [34]. Another explanation for the absence of an effect of PBDs on HDL-C in the current study is that the design distinguishes between various PBDs, whilst other studies may have had increased power to detect differences in biomarkers by combining vegan and vegetarians into the same dietary category for analyses.

Nutrient composition of PBDs may explain differences in plasma lipids observed across diet groups. Although overall fat intake was comparable, when compared to RMEs, vegans and LOVs had significantly lower intake of saturated fat, trans-fat, and cholesterol and higher polyunsaturated fats (vegans only). LOVs had significantly lower trans-fat intake and PVs and SVs had comparable intakes to RMEs. These outcomes are aligned with previous larger observational studies with alike characterisations of vegan and LOV dietary patterns. Such studies observed vegans to have the lowest dietary intake of saturated fat, trans fats and cholesterol and highest intakes of PUFAs compared to a regular meat-eating diet [50–53]. Furthermore, analyses of daily food group intake provided deeper insight into how dietary habits contribute to dietary fat profiles. PBDs in the present study had significantly higher intakes of legumes/nuts as their source of protein rich foods, which are predominantly richer in dietary soluble fibre, unsaturated fats, whilst consuming minimal/nil animal-based protein, inherently rich in saturated and trans fats. It is well documented that dietary saturated and trans fats have been shown to increase LDL-C and TC concentrations, therefore increasing the risk of atherosclerotic CVD [54]. PUFAs, on the other hand, are known to benefit heart health by lowering TG and LDL-C concentrations through mechanisms such as reducing systemic inflammation, improving endothelial function, and acting as an antiatherogenic agent [55, 56]. RMEs had the highest saturated fat intake, lowest PUFAs intakes and highest plasma lipids levels. In contrast, vegans had the lowest saturated fat intake, highest PUFAs intakes and lowest plasma lipids.

Intake of n-3 PUFA are known to specifically reduce TG concentrations. As expected, intake of fish and seafood, and LC n-3 PUFAs was significantly higher among those adhering to a PV dietary pattern compared to all other groups (except for RMEs for LC n-3 PUFAs). However, levels of consumption were moderate (half a serve a day) which explain why a dietary pattern, although higher in n-3 PUFAs rich foods did not significantly lower TG concentrations. In addition, RMEs had the highest number of participants supplementing with EPH/DHA compared to PBDs. Although appropriately adjusted for, this may have an influence on crude TG concentrations. Lastly, intake of dietary fibre can also play a key role in reducing absorption of saturated and trans fats, which therefore lowers LDL-C and TC and has been proved to reduce risk of CVD [57]. Soluble fibre lowers lipid levels by binding to cholesterol particles therefore preventing absorption [57]. Moreover, soluble fiber can also bind to bile acids which results in a reduction of cholesterol content within the liver cells, upregulation of LDL receptors and clearance of circulating LDL-C [58]. In the present sample, compared to RMEs, individuals adhering to a PBD consumed more dietary fibre, vegetables, fruit, legumes/nuts and fruits which are rich in soluble fibre, which is supported by previous large cross-sectional and prospective studies [50, 51, 53]. Detailed collection of micro and macro nutrient compositions related to intake of dietary fats and fibre helps to cement our understanding of the independent effect of dietary pattern on cardiometabolic risk factors.

In the current study, compared to RMEs, those adhering to a vegan dietary pattern were associated with favourable glycaemic indices characterised by significantly lower FBG, HbA1c, and insulin. In addition, LOVs had significantly lower FBG compared to RMEs. PBDs have been offered as a strategy for managing glycaemic control in previous literature [24]. A meta-analysis of six RCTs following vegetarian dietary patterns for ≥ 4 weeks found they were associated with significant reduction in HbA1c and a non-significant reduction in FBG [59]. Another meta-analysis investigating the effect of replacing animal protein with plant protein in RCTs with a median follow up of 8 weeks reported a significant reduction in HbA1c, FBG and insulin [60]. In addition, a meta-analysis of fourteen observational studies found a vegetarian (including vegans) dietary pattern was inversely associated with risk of T2D compared to non-vegetarians [61]. Very few studies have investigated the effects of PBDs, inclusive of PVs and SVs on glycaemic indices and risk of T2D. The AHS-2 study found vegans, LOV, SV but not PV were associated with a substantial and independent reduced risk in T2D after a two year follow up period [28]. The same results were observed in the EPIC-Oxford study, except for PVs, which had a follow up period of eighteen years [62]. Moreover, an Australian-based study using the same definition of PBDs as the current study, found prevalence of impaired glucose tolerance was lower in vegans, LOVs, SV, and PVs, compared to RMEs [15]. Both meta-analyses and similar observational studies corroborate results seen in the present study which demonstrate those following a vegan and or LOV dietary pattern had lower FBG and HbA1c compared to RMEs.

Our study demonstrated that the vegan dietary pattern had lower insulin levels when compared to RMEs. Findings are corroborated by a previous, age and sex-matched cross-sectional study which reported that a vegan dietary pattern had lower insulin and HOMA-IR, however, significance for LOVs was lost after adjustment for confounders such as BMI, physical activity, and alcohol consumption [63]. Furthermore, two separate RCTs reported both vegans and LOVs interventions reduce insulin resistance (HOMA-IR) and insulin levels when compared to an omnivorous intervention after 16 weeks and 24 weeks, respectively [64, 65]. Few studies have explored the relationship between PVs and SVs and insulin levels. Results from a small cross-sectional study found individuals who maintained a long-term SV had lower insulin concentrations and insulin resistance (HOMA-IR) when compared to non-vegetarians [66]. This study strengthens the developing recognition of vegan and LOV dietary patterns as effective tools in the prevention and management of glycaemic indices and potential improvement in insulin sensitivity which are key risk factors for CVD [16, 67]. There is not enough research to conclusively define influence of SV and PV dietary patterns on glycaemic indices and insulin sensitivity, although emerging findings are favourable. Future longitudinal studies evaluating prevalence of T2D, glycaemic indices, and insulin resistance across various PBDs, inclusive of SV and PVs are warranted to substantiate outcomes observed in the current study and small pool of previous literature.

Nutrient composition across dietary patterns may be linked to differences in glycaemic indices. Vegans, LOVs and SV had significantly higher carbohydrates (EN%) than RMEs, and vegans also had significantly higher intake of starch. However, as vegans had significantly lower glycaemic indices than RMEs, it is apparent that consumption of food groups as opposed to individual nutrients may better explain observed differences. PBDs, specifically vegans had higher intake of legumes/nuts, fruit and lower intakes of discretionary choices characterised by nutrient poor foods high in added sugars, energy, salt, saturated fats, compared to RMEs [68]. Evidence suggest that a dietary pattern rich in fibre and wholefood groups and lower in processed meat, red meat and discretionary choices including sugar sweetened beverages can reduce risk of T2D [69]. Added sugar foods and high glycaemic rich carbohydrate are rapidly absorbed after consumption leading to an increase in blood glucose and insulin responses, which if sustained over time can lead to glucose intolerance and insulin resistance, corner stones for T2D [70]. Previous literature not only confirms the ability of PBDs to favourably modulate glycaemic indices and risk of T2D, but also suggest that a vegetarian dietary pattern may have greater capacity to improve insulin sensitivity compared with a conventional diabetic diet, characterised by lower carbohydrate and higher protein intakes [65].

The novelty of this study is such that it is the first ever Australian-based study to purposefully recruit individuals habitually following several types of PBDs from the community to evaluate health status and risk of cardiometabolic biomarkers. Majority of the previous literature on PBDs are from secondary analyses, which have not specifically recruited individuals habitually following PBDs from the community, therefore, can only evaluate indexes, for example healthy and unhealthy plant-based indexes. This study design did not result in retro-fitting participants into indices which allows for better translation to clinical practice and nutrition policy interpretation. Results contribute to existing literature by providing distinguished differences between various PBDs and regular meat-eating diets and their association with key risk factors for CVD and T2D, not previously explored. Outcomes are underpinned by comprehensive investigation of dietary patterns, a primary lifestyle risk factor for these chronic conditions.

Strengths include use of validated and dietitian administered detailed dietary collection tools which provide comprehensive qualitative and quantitative nutrient intake data fundamental to reflect the complex nature of human dietary patterns [38]. Second, the duration in which participants were following dietary patterns was long enough to exert an effect on primary outcomes. Third, several potential covariates were controlled for to avoid confounding and therefore appropriately examine the association between PBDs and lipaemic and glycaemic indices whilst considering other lifestyle factors. Lastly, the level of difference between the vegan dietary pattern and RMEs for LDL-C and HbA1c was deemed clinically significant as defined by a ≥ 10% and ≥ 0.5% difference, respectively, and therefore translational and practical for use in health care and nutrition policy [71]. However, several limitations need to be considered. Being a cross-sectional study, findings should be interpreted with caution and no inferences on causation can be employed. While some data were self-reported, all questionnaires were reviewed by study investigators with participants, and use of validated data collection methods such as the AES^®^ FFQ [36] and IPAQ were used [44]. Although adequately powered, the sample size is modest and future larger studies using similar validated dietary collection methodologies are warranted.

## Conclusion

In conclusion, this cross-sectional study of healthy middle-aged adults found individuals adhering to a vegan dietary pattern had favourable plasma lipids and glycaemic indices compared to regular-meat dietary patterns characterised by significantly lower concentrations of TC, LDL-C, non-HDL-C, TC/HDL ratio, FBG, HbA1c and insulin. Other PBDs had a moderate association; SVs had lower LDL-C and non-HDL-C; and LOVs had lower FBG. HDL-C and TG were comparable across plant-based and regular meat-eating dietary patterns. This study highlights the importance of detailed dietary collection methodologies to effectively evaluate links between dietary patterns, metabolic biomarkers, and overall cardiovascular health. Findings from this study have direct application to clinical practice, suggestive of at a minimum, the consideration of plant-forward dietary patterns for the assistance of managing elevated blood lipid and/or glycaemic parameters among those at higher risk of CVD. In addition, outcomes warrant further consideration of PBDs to be incorporated alongside dietary advice relating to cardiometabolic disease risk reduction, as well, as among national population-based dietary guidelines. Larger forthcoming prospective studies utilising similar methodologies to define and evaluate PBDs, inclusive of PVs and SVs, are warranted to substantiate findings and further examine effects of PBDs on the development of chronic diseases.

## Electronic supplementary material

Below is the link to the electronic supplementary material.

Supplementary Material 1

## Data Availability

No datasets were generated or analysed during the current study.

## Abbreviations

AHS-2: Adventist Health-2 study
CVD: cardiovascular disease
DHA: docosahexaenoic acid
EPA: eicosapentaenoic acid
FBG: fasting blood glucose
HbA1c: haemoglobin A1C
HDL-C: High-density lipoprotein cholesterol
HOMA-IR: Homeostatic Model Assessment for Insulin Resistance
IPAQ: International Physical Activity Questionnaire
LDL-C: low-density lipoprotein cholesterol
LOV: lacto-ovo vegetarian
METs: metabolic equivalent of tasks
MetS: metabolic syndrome
LC N-3 PUFAs: long chain omega-3 poly-unsaturated fatty acids
PBD: plant-based diet
PV: pesco-vegetarian
RCT: randomised controlled trial
RME: regular meat-eater
SV: semi-vegetarian
TC: total cholesterol
TG: triglycerides
T2D: type 2 diabetes mellitus
EN%: percentage total energy intake
