# Supplementary material for: Plasma lipids and glycaemic indices in Australians following plant-based diets versus a meat-eating diet
Source: Lipids Health Dis. 2024 Oct 26;23:348. doi: 10.1186/s12944-024-02340-5 (PMC11514886; doi:10.1186/s12944-024-02340-5)
Supplement: Supplementary file 1 — Supplementary Material 1 [file 12944_2024_2340_MOESM1_ESM.pdf]

Supplementary Table 1. Adjusted means, 95% CIs and percentage differences in lipid levels and glycaemic indices of plant-based diets compared to regular meat-eating diets.

	**Vegan (n=48)**	**Lacto-ovo vegetarian (n=47)**	**Pesco-vegetarian (n=46)**	**Semi-vegetarian (n=44)**	**Regular meat eater (n=45)**
**Lipid levels**					
TC (mmol/L)	4.8 (4.56, 5.04)	5.37 (5.13, 5.60)	5.45 (5.19, 5.70)	5.20 (4.99, 5.41)	5.57 (5.30, 5.85)
%	**14.85***	3.66	2.18	6.87	-
TG (mmol/L)	1.01 (0.87, 1.15)	1.16 (1.01, 1.31)	1.01 (0.81, 1.21)	1.09 (0.96, 1.22)	1.10 (0.96, 1.23)
%	8.53	5.31	8.53	0.91	-
LDL-C (mmol/L)	2.79 (2.58, 2.99)	3.27 (3.06, 3.48)	3.37 (3.15, 3.59)	3.09 (2.91, 3.28)	3.50 (3.26, 3.74)
%	**22.57***	6.79	3.78	**12.44**	-
HDL-C (mmol/L)	1.54 (1.44, 1.64)	1.59 (1.50, 1.68)	1.62 (1.50, 1.74)	1.61 (1.51, 1.71)	1.57 (1.47, 1.67)
%	1.93	1.27	3.13	2.52	-
Non-HDL-C (mmol/L)	3.25 (3.03, 3.47)	3.80 (3.57, 4.02)	3.83 (3.57, 4.10)	3.59 (3.39, 3.80)	4.00 (3.75, 4.25)
%	**20.69***	5.13	4.34	**10.80**	-
TC/HDL-C ratio (mmol/L)	3.18 (2.94, 3.42)	3.53 (3.31, 3.76)	3.55 (3.25, 3.84)	3.54 (3.19, 3.89)	3.67 (3.44, 3.89)
%	**14.31**	3.89	3.23	3.61	-
**Glycaemic indices**					
FBG (mmol/L)	4.56 (4.41, 4.70)	4.51 (4.38, 4.64)	4.71 (4.56, 4.86)	4.64 (4.48, 4.81)	4.85 (4.68, 5.02)
%	**6.16**	**7.26**	2.93	4.43	-
HbA1c (IFFC, mmol/mol)	31.8 (31.1, 32.6)	33.3 (32.5, 34.1)	33.0 (32.2, 33.8)	33.1 (32.4, 33.8)	33.7 (33.0, 34.4)
%	**5.80**	1.19	3.01	1.80	-
HbA1c (NGSP, %)	5.07 (5.00, 5.14)	5.20 (5.13, 5.27)	5.17 (5.11, 5.24)	5.19 (5.13, 5.25)	5.24 (5.18, 5.31)
%	**3.30***	0.77	11.34	0.96	-
Insulin (mU/L)	5.26 (4.36, 6.16)	6.55 (5.18, 7.91)	6.00 (5.47, 6.52)	7.11 (5.95, 8.27)	7.02 (6.06, 7.98)
%	**28.66**	6.93	15.17	1.27	-

Data is presented as adjusted means, 95% CIs and percentage differences. Multivariate regression analyses was used to adjust the model for age (years), sex (female, male), physical activity level (MET/week), total energy intake (kJ/day), duration of dietary pattern (years), alcohol intake (g), smoking status (yes, no), level of education (higher education yes, no), BMI (kg/m^2) as a mediator and the addition of EPA/DHA supplement use (yes, no) for lipid levels. Bold values indicate statistical significance ($P<0.05$). *P*-values marked with an asterisk meet the Bonferroni corrected alpha ($P=0.001$). EPA, eicosapentaenoic acid; DHA, docosahexaenoic acid; FBG, fasting blood glucose; HbA1c, haemoglobin A1C; HDL-C, high-density lipoprotein cholesterol; LDL-C, low-density lipoprotein cholesterol; TC, total cholesterol; TG, triglycerides.
